# Mentees’ Perspectives on Mentorship and Sponsorship in Academic Anesthesiology: Findings from a Single-institution Focus Group

**DOI:** 10.1097/ao9.0000000000000033

**Published:** 2026-09-30

**Authors:** Anne L. Donovan, Seyedeh Ala Mokhtabad Amrei, Ting Guo, Joyce Chang, Jina L. Sinskey, Oana Maties, Rachel Schwartz

**Affiliations:** 1 Anne L. Donovan, M.D., Joyce Chang, M.D., Jina L. Sinskey, M.D., and Oana Maties, M.D.: Department of Anesthesia and Perioperative Care, University of California, San Francisco, San Francisco, California.; 2 Seyedeh Ala Mokhtabad Amrei and Ting Guo, B.S., B.A.: University of California, Berkeley, Berkeley, California.; 3 Rachel Schwartz, Ph.D.: Department of Anesthesia and Perioperative Care, University of California, San Francisco, San Francisco, California; Division of General Internal Medicine, Department of Medicine, University of California, San Francisco, California; Center for Health Equity in Surgery and Anesthesia, University of California San Francisco, San Francisco, California; Philip R. Lee Institute for Health Policy Studies, University of California San Francisco, San Francisco, California.

## Abstract

Mentorship and sponsorship are important to career advancement and sustainability within academic anesthesiology, but unique challenges contribute to the gap that exists between optimal and actual mentorship and sponsorship. In this article, focus group data from a study conducted among 38 academic anesthesiology faculty members in a single department, stratified by self-identified gender and career stage as well as identity-specific groups, formulate three themes: (1) elevating high-impact mentorship, (2) formalizing sponsorship infrastructure, and (3) implementing a structured program. These themes and their subthemes are examined within a framework of questions that aid in constructing meaning and moving toward practical application. The results of the thematic analysis are well-supported by the literature presented in the final section of the article and also build on current literature by suggesting potential design principles for mentorship and sponsorship structures synthesized directly from academic anesthesiology faculty across diverse career and life stages and identity-specific groups.

Mentorship and sponsorship are critical constructs for career advancement and sustainability in academic medicine. Mentorship is known to benefit mentees, mentors, institutions, and academic fields at large^1,2^ and to influence individual career development, personal growth, and academic productivity.^2–4^ Further, mentorship and mentorship programs have been tied to retention in academic medicine.^5,6^ Sponsorship is a distinct yet related concept that is also crucial to establishing momentum in an academic career path.^7,8^

A mentor is traditionally defined as an experienced individual who provides guidance, support, and advice to a less experienced individual (*i.e.*, the mentee) to further their development in one or more areas.^9^ Mentorship is the process in which mentors and mentees engage primarily for the benefit of the mentee; however, mentor–mentee relationships are increasingly recognized as bidirectional, meaning that both parties contribute to and receive some benefit from the relationship.^1^ A mentor may serve other simultaneous roles in addition, including role model (*i.e.*, a source of inspiration often exemplifying qualities one wishes to emulate), advisor (*i.e.*, an authority figure who assists in making informed decisions), sponsor (*i.e.*, an experienced person who provides access to resources or opportunities and/or advocates for more junior individuals using their own influence or capital), and/or coach (*i.e.*, one who provides support or guidance to a learner toward specific goals, often on a shorter term basis).^1^

Unique challenges in academic anesthesiology systematically disadvantage the process of mentorship and sponsorship in the field. There is a growing body of literature documenting the isolated nature of anesthesia practice;^10^ limited access to mentors and sponsors, particularly for women and underrepresented individuals;^11,12^ lack of institutional support structures;^12^ and time constraints, which disproportionately affect women.^12^ However, many of the existing studies on this subject fail to account for individual variations in mentorship needs and do not provide specific guidance for underrepresented faculty.^13^ While studies have described a gap between desired or optimal mentorship and real-life mentorship in academic anesthesiology,^12,14–16^ more work remains to develop practical solutions to these problems. Previously proposed solutions include integrating mentorship, sponsorship, and allyship into the culture of anesthesiology at the departmental and organizational levels to improve belonging and encourage retention^11^ and to advance gender equity within anesthesiology leadership structures.^17^ However, prior studies lack the detail needed to serve as a practical guide for implementing mentorship and sponsorship structures that are accessible, adaptable, and scalable within academic anesthesiology.

In this article, faculty participating in a series of focus groups conducted within a large academic anesthesiology department describe features of high-impact mentorship and sponsorship and mentorship programs in academic anesthesiology. Faculty perspectives are then related to the broader literature on this topic. This work builds on current literature by suggesting design principles for mentorship and sponsorship structures that are synthesized directly from academic anesthesiology faculty across diverse career and life stages and identity-specific groups.

## Approach to Data Collection

The establishment of the focus group, participant selection, and data collection methods used for this Special Article were described previously in our recent publication.^18^

### Participant Selection

Focus group participants were faculty members in the department of anesthesia and perioperative care at a large urban academic medical center, which includes an academic tertiary care center and a county hospital with a separate direct leadership structure. Faculty work at six distinct clinical locations, which include a tertiary care academic referral hospital; a hospital dedicated to the care of women, children, and cancer patients; an ambulatory surgery center; an ambulatory orthopedic surgery center; an orthopedic inpatient hospital; and a public county hospital and Level 1 trauma center serving a socioeconomically underserved population. A high degree of inter-group variability in clinical practice settings, subspecialties, and academic interests exists among participants. Faculty volunteered to participate in a focus group by completing an online form presented at the end of an optional department-wide faculty survey.^19^ The form allowed volunteers to self-select appropriate focus group(s), indicate a preferred order for group assignment, and provide consent to participate. Based on form responses, participants were assigned to one of nine focus groups (table 1): Early career women and men, mid-career women and men, established-career women and men, parents, researchers, and underrepresented in medicine. Grouping by career stage allowed for targeted mentorship and career development questions and for candid conversation between near-peers, while grouping according to gender allowed for discussion of perspectives on the impact of gender on career trajectory. Specific stressors facing vulnerable populations in medicine were discussed within groups for parents, researchers, and underrepresented in medicine individuals; no specific career stage guidance was provided for self-selection into these groups. Because of the inductive aim of the study, saturation was not the criterion on which focus group size was based; however, the number of focus groups conducted was sufficient to achieve code saturation.^20^ Early career was defined as assistant professor or instructor rank, mid-career was defined as associate professor rank, and established career was defined as professor rank based on the specific promotion process at the study institution. This study was granted exempt status by the University of California, San Francisco Institutional Review Board (IRB # 23-39253). The manuscript was prepared according to the Standards for Reporting Qualitative Research (SRQR) checklist.^21^

**Table 1.** Focus Group Identifier Labels

Focus Group	Label	Number of Participants	Number of Years on Faculty, Average (Range)
Early career women	FG1	3	2 (1–3.5)
Early career men	FG2	5	2.7 (0.5–4)
Mid-career women	FG3	4	9 (8–10)
Mid-career men	FG4	4	8 (6–10)
Established-career women	FG5	3	17 (14–20)
Established-career men	FG6	6*	19.4 (17–21)
Parents	FG7	3	7.2 (5.5–9)
Researchers	FG8	6	10.5 (5–17)
Underrepresented in medicine	FG9	4	5.75 (2–8)

* Six total participants in two separate FG (2 and 4 participants).

FG, focus group.

### Focus Group Process

For each focus group, Zoom scheduling software (version 6.3.4, Zoom Video Communications, Inc., USA) was used to select a 1-h meeting time that maximized the number of attendees per group. Each focus group was conducted over Zoom using semi-structured interview guides developed by an established-career female anesthesiology and critical care faculty member (A.L.D.), a mid-career female anesthesiology and critical care faculty member (J.C.), an established-career female anesthesiology faculty member (O.M.), and a female qualitative health services researcher with expertise in physician well-being research (R.S.). Questions were informed by a prior department-wide survey that had been conducted to understand the experiences of faculty members within the department^19^ and were pilot tested and refined with clinical colleagues not involved in the focus groups. General categories covered by the interview guides include: demographics; faculty experience; allyship, mentorship, and sponsorship; and perceived needs for a structured departmental mentorship program. Each interview guide was tailored slightly to the focus group of interest (Supplemental Digital Content 1: https://links.lww.com/ALNO/A28). Focus groups were conducted by one of two members of the research team (A.L.D. and R.S.) with support from a third team member (O.M.). The interviews were transcribed, deidentified, and imported into Dedoose (version 10.0.35, SocioCultural Research Consultants, LLC, USA). Only the coding team had access to the transcripts.

Of the interviewers, A.L.D. was known to most focus group participants as a department faculty member; however, because she did not hold any leadership positions related to participants’ clinical or academic work, the relationship was collegial and nonhierarchical. R.S. was known to most participants in the research focus group and similarly had a collegial, nonhierarchical relationship with participants. There were four members of the coding team: two female premedical students (T.G. and S.A.M.A), A.L.D., and R.S. Measures taken to reduce bias during the coding process include involvement of both clinical (A.L.D.) and nonclinical (R.S.) faculty in addition to researchers external to both the department and academic medicine (T.G., S.A.M.A.), which allowed for discussion of the transferability of ideas raised. Reflexivity was employed in the discussion of potential biases arising from lived experiences within the team, thereby challenging assumptions made throughout the coding process. The team used an inductive thematic analysis approach^22^ and followed a constructionist paradigm. The entire coding team met weekly for 3 months to establish and refine a codebook. To ensure intercoder reliability, each transcript was reviewed by at least two coders, with any discrepancies resolved through full-team discussion and consensus.

## Participant Demographics

Thirty-eight out of 51 faculty members who volunteered for the study ultimately participated in a focus group. Constraints in scheduling a group meeting in this population prevented all volunteers from participating. Each group had three to six participants, with a median of four (table 1). A single focus group was conducted for each group except for the established-career men group, which required two separate focus groups because of scheduling constraints (two and four participants per group, total six participants). Data were pooled for this group for the purpose of reporting. Nineteen participants were women and 19 were men. Thirteen participants were early career (average 2.4 yr on faculty, range: 0.5 to 4 yr), 12 were mid-career (average 8 yr on faculty, range: 6 to 10 yr), and 12 were established-career (average 18 yr on faculty, range: 14 to 21 yr) (table 1). Additional demographic details were previously reported.^18^

## Thematic Analysis

Across focus groups, participants identified mentorship and sponsorship as vital for career fulfillment and retention, particularly during vulnerable transitions. Despite their acknowledged importance, participants noted the “freeform” nature of current practices, and some also shared negative or suboptimal experiences with mentorship and sponsorship. Both positive and negative experiences were integrated to propose structures that participants would find helpful for formalizing mentorship and sponsorship. Consequently, three themes emerged: (1) elevating high-impact mentorship, (2) formalizing sponsorship infrastructure, and (3) implementing a structured program (fig. 1). Theme 1 had two subthemes that characterize high-impact mentorship (*tailored mentorship* and *mentorship relationship*), theme 2 had three subthemes that advance sponsorship on an individual and departmental or institutional level (*network-based sponsorship seeking*, *intentional sponsorship practices*, and *departmental structures and culture as sponsorship enablers*), and theme 3 had five subthemes that offer participant-proposed considerations for implementing a structured program (*accommodate barriers*, *allow voluntary participation*, *facilitate intentional mentor–mentee matching*, *provide meeting structure*, and *align with professional development initiatives*). These themes and subthemes are explored further below using questions as descriptive prompts. Illustrative quotes corresponding to each theme, subtheme, and question are listed in table 2.

**Table 2.** Themes and Subthemes from Focus Group (FG) Data

Theme	Subtheme
**Theme 1:** **Elevating high-impact mentorship**	
*Why is mentorship important in academic anesthesiology?*	
“[On the Merits and Promotions Committee] we’re always finding that junior faculty…have one year to [create their first advancement packet]. And it’s really hard to get anything meaningful on that CV.” (FG3) “I think [at mid-career] you get to a point where…you’ve gotten some leadership roles, and now there’s a huge void of like, okay, well, here I am…[where] do I go now? I’m supposed to be the mentor. Like, who do I talk to now? And now I even feel maybe slightly more lost but a little less overwhelmed, I guess I would say.” (FG3) “I think it’s been an interesting evolution, because I feel like the mentorship I need now as a mid-career faculty is very different because I’m relatively independent with my research projects themselves. So it’s more about kind of big picture career advising or asking about opportunities or career direction like planning five to 10 years out from now and not so much a day-to-day mentoring relationship.” (FG8) “I sort of divide it into apprentice versus consultant kind of roles. Often early in your career, it makes a lot of sense to…be the apprentice to somebody who is successful and has the attributes of a scientist that you want and some topic that you’re interested in and a good grasp of your career path or an outside career mentor. And then as you become more and more self-determined as a scientist, people start to move from a mentor role into more like a consultant where you have a specific goal for them in mind and it might be time limited or it might be longitudinal.” (FG8)	Mentorship tailored to career stage
“I don’t think there’s any one perfect mentor.” (FG4) “I think the junior faculty in research should have two mentors. They should have a career structure mentor and a content mentor that can provide them with the substance of what they’re studying or can help them with that.” (FG8) “I feel like our work situation [as parents and anesthesiologists] is unique in a lot of ways, like the unpredictability of life,…scheduling, how early we start, like how stressful our jobs are, like the academic pressure to [produce and] contribute to the university in some way…it would be really great to talk to somebody, see if they have life advice, like how they balanced it, for example, during like the early years when their kids were little and maybe needed them more before they were, I don’t know, a little bit more self-sufficient in the teenage years.” (FG7) “…it doesn’t have to be a person that is from your same background. Like we all have different mentors, but perhaps it can be a person that had experienced similar things or can share with you how they have done certain things so they can mentor you on that.” (FG9)	Mentorship tailored to domain
*How should a mentee identify and select an appropriate mentor?*	
“For me, mentorship has always sort of happened organically…I guess mentors have sort of come and gone for me to some extent. But… it’s always sort of been just connecting with someone on a professional level and then finding that there’s some shared interests. [I look at the] career of other people that I would like to emulate. I’ve sort of used that [as a] barometer [to judge] whether I think [we] would align, and then [I approach] people kind of on my own and [see] if we can have a working relationship, if things click.” (FG4) “[I think it’s important that it’s] at your discretion who your mentor is, number one, and then number two, that in some ways that that person isn’t…evaluating you. …I would want my mentor to be somebody who can support me in a time of need or who sees me at my best…So the mentor being somebody who kind of has no consequence over your advancement would be important to me at least.” (FG9) “And the individual who I initially chose was the person that I felt like I had the most connection with and was able to, you know, provide a really good understanding of the department and help me, you know, find the right people faster. And now that has evolved. And so that…formal process has just turned into more of a colleague friendship and not necessarily mentorship. I think…it’s hard to truly know how and when that relationship will truly form. And so I’ve kind of let it happen organically and through my personal interests, individuals have been very kind to include me in conversations.” (FG1)	Mentorship relationship: organic relationship
*What essential characteristics should a good mentor possess?*	
“I think the availability [is one quality that is] really important, because [not all people] have the time to spend mentoring people – [and] the more junior you are, I feel like the more hands-on you have to be. … I think somebody who like can see a longer-term vision for your career and not just see you as like a short-term cog in the wheel, like helping to get their projects done, but they actually see how your overarching career path might fit within their research program [can help you become] independent at the end of the day.” (FG8) “It’s very vulnerable for us to talk about [childbearing] openly with a mentor, [but] if it’s somebody that you really trust, [who is invested in you, and who can] share about their hardships because they [know you well, that’s] the other piece to all of this as well.” (FG3) “… I really have had like a couple very positive mentorship relationships, but they have not looked like me. …But [it’s more about] caring about the person’s success and really like feeling like they’re invested in you [and your career success].” (FG9) “I think mentorship…works the best when the mentor considers the mentee as a whole, as like a whole person. …It’s not about what [the mentor wants]. It’s about getting [the mentee] where they want to be and like working within the context of their life and their training.” (FG7) “Whoever the mentors or the sponsors are, I think they need to be trained. Because I think… in the past [my mentors have also been] struggling with the same things I’m struggling with. …What I needed is somebody who looks at like sort of my time that I have available, looks at my career goals, and is like, okay, these things that you’re doing are promotable and are going to get you there. These things that you’re doing are not promotable. Don’t waste your time on it. Or here’s how you kill like three birds with one stone with this one project.” (FG7)	Mentorship relationship: effective mentors
* What should a mentor expect from a mentee?*	
Mentee perspectives: “I have been able to receive like good mentorship..., but I certainly think that it was from my own sort of like reaching out, being aggressive about…finding mentors like myself and really like reaching out, having like multiple meetings…[it’s a very active process], it doesn’t just kind of naturally happen.” (FG9) “As mentees, we also have the responsibility to reach out to our mentor and not just [make it] their responsibility…And if we have goals, it’s also up to us to…work to get there.” (FG1) “One thing I’ve realized is an important part of like a mentor-mentee relationship also relies on the mentee. And I’m not sure, to be honest with you, like how much I realized that when I was the mentee…you have to kind of initiate things and engage things. …Is it my fault that I wasn’t like mentored properly because I didn’t like seek things out? Maybe like no one told me I needed to do that.” (FG5) “…you make such a good point about not being a good mentee. I mean, I was terrible, right? When we were assigned mentors, I didn’t bother really connecting. …Half the time they would say something [and I would think], ‘no, actually, that’s not what I’m thinking at all,’ but I wouldn’t necessarily [speak up]. I would just smile and shake my head and move on. So…there is some responsibility on the side of the mentee [to make the relationship meaningful].” (FG5) Mentor perspectives: “I think about this a lot as someone who is trying to be a better mentor…I know how busy this young faculty member is. I don’t wanna bother them. I don’t want to force mentorship on someone because I know just like maybe how overwhelming that might be like, oh, now I have another meeting to go to or I have to like show this person I’m doing something. So I think that the ask [from the mentee] for time or space [is] huge because otherwise I think mentors too are a little bit wary of reaching out and scheduling meetings with someone who already doesn’t have the bandwidth to do that.” (FG3) “On the other hand, I think that the relationship is really a partnership and that you should both be benefiting from it. If one is only giving and the other is taking, then it feels as if you’re being used or exploited. I like to think of [mentor and mentees as peers] so that we’re working collaboratively together.” (FG8) “[Mentees need to be persistent, sometimes it takes] several emails to remind [the mentor]. …So yeah, just persist and pursue is something [to keep in mind]…you just need to put in time to keep contacting them.” (FG8) “An appreciation for the extent to which mentoring someone [in research] is a tremendous investment for the mentor as well in a way that a clinical mentorship probably isn’t. If I agree to mentor someone in a scientific topic, I expect to spend 400 hours. Especially if it’s an apprenticeship-type scientific mentorship, like we may work one-on-one 20 hours a week for the foreseeable future, that that kind of time investment is necessary for a lot of people, particularly early in their career.” (FG8) “I’m a mentor to people who are going through the merits and promotions process…every year. And most people do really well, most people are on target, but I have to chase some people down, like update your CV, update your CV, like you’re gonna miss the deadline. [A few people who have not completed their packet] may not get advanced. They may not get their merit because they have not done the work.” (FG6)	Mentorship relationship: proactive mentees
* How can an effective mentorship relationship be developed, nurtured, and sustained?*	
“[The mentor] having themselves like a growth mindset of being a good mentor. Like the coming back and saying like, oh, was that helpful?...What else can I do? [Letting you know] I’m thinking about your career and I have a good idea of like what you should focus on…shows investment and [that] they’re actively trying to themselves become a good mentor is [important to developing a relationship].” (FG9) “[Both mentors and mentees should] really want to do it. Like, I don’t think anyone can be forced into this, you know, otherwise it’s going to fall apart before it even starts. Just make sure that you have people that really actually care and really actually want to do this.” (FG9) “I think the relationship that works really well is when [the mentee] is self-motivated and self-driven to just do the work because it can be super exciting to watch somebody kind of take off that way. [There needs to be] buy-in from both parties, but I think both parties do have to lean into it a little bit.” (FG5)	Mentorship relationship
**Theme 2: Facilitating organized sponsorship**	
* How does sponsorship complement mentorship?*	
“[Someone with a sincere interest in sponsorship explained to me in a granular way how] promotions work, but not just like what’s on the piece of paper [but also the hidden curriculum] – you’ve got to sign up for these organizations, got to go to ASA, you know, like those little things that…when you’re so stuck in the weeds of clinical work and you can’t really think about anything else except your next case, like someone’s there to shine a light overhead.” (FG9) “[At mid-career], you do sort of need to start developing at least some degree of recognition outside of the institution, and I think making that transition is challenging. [You rely on opportunities to] raise your recognition a little bit outside the institution.” (FG4) “And I think many people appreciate that on a sort of a, one-on-one level, [our Division Chief] had a large presence in [our subspecialty] world and, you know, got me a couple of speaking opportunities…and sort of helped me sort of get a toehold in some ways early on.” (FG6)	
* What intentional practices are recommended for seeking and offering sponsorship?*	
“…Especially [in my subspecialty] group, you have a lot of people that have been helpful in knowing what your interests are and kind of presenting you with opportunities as they come along…So I think just having colleagues that also have your best interest has been really helpful.” (FG4) “If you’re willing to put yourself out there and put in the extra work, I think people notice it and are willing to bring you on board to different teams or different areas of leadership.” (FG6) “I guess sponsorship takes time and trust. It’s not something [that is] freely available. We have to work hard to get sponsorships.” (FG5)	Network-based sponsorship seeking
“I think a degree of [structured] sponsorship would be helpful, maybe even in a very non-threatening way, [for example], some sort of audit of your CV and career, just someone more senior to kind of really sort of…do a deep dive and make sure that you’re on the right path.” (FG4) “For young faculty, I think sponsorship is really important…we do get [notifications about] different opportunities and positions that are popping up, sent to our emails. A lot of them get lost in the weeds, you know? It would be great if there was…a streamlined place [that compiled available] projects or like, you know, this person’s doing this, or…if anyone is interested and wants to look for a project, like everything’s listed here, reach out to these people and these people would actually be willing to sponsor you to kind of help you do that. I think that that would help in terms of advancement. I think it would help allow younger faculty find different things to get involved in to see what they settle into, see what they like and what they don’t like and to carve their own path, you know?” (FG9) “Every time something has come up and I was interested, I felt like the door was open, or people were open to me taking roles and leadership roles. So I feel very fortunate that I’ve had the opportunities that I’ve had, and I hope that I can create that for other people coming after me.” (FG6)	Intentional sponsorship practices
“Part of mentorship and sponsorship is, you know, getting out of the way, right?...And sometimes that requires leaders above to clear the lane. …You can stay forever…in positions of leadership and frankly power and influence. [Good mentorship] is not just [to] shove people into opportunities, but [to help them] find new opportunities, and also to help clear the way for younger generations, junior faculty.” (FG6) “[After] three years [in a leadership position], you’ve done your legacy, pass it on. Why are you holding on to it? [Individuals could be encouraged to move out of] that position, because that should not be where your attention should be at that point in your career.” (FG5) “I think it’s about making opportunities possible and then also giving the time to run with those opportunities, right? Like it’s really hard to do sort of leadership programs or any training or anything further if there’s not the bandwidth in your clinical schedule to make that happen. …If the department really wants to move things in a different direction, I think that there has to be actual dedication to creating more space and time for it.” (FG3) “Could there be a way to have the department have kind of a smaller, a small or a large selection of kind of much more quickly rotating responsibilities attached with something, non-clinical time or recognition or whatever that could be…distributed in a way that would make people feel like they’re getting what I guess I’m calling structured sponsorship?” (FG4)	Departmental structures and culture as sponsorship enablers
**Theme 3: Implementing a structured program**	
* How should an institution or department establish, govern, evaluate, and continuously improve a formal mentorship program?*	
“[My division Chief and I] have tried for two or three months to just have a meeting one-on-one. And it never happened because of our crazy schedule. Somebody is either post-call or, you know, we are running different rooms. And so we never got to sit together and talk.” (FG1) “I think it’s something that’s extra for people to do, whether you’re a mentor or a sponsor. And it’s hard, even if you make that someone’s job, like the people you’re trying to mentor or sponsor have an overwhelming, very busy job as a physician in the [current time].” (FG6) “I think that there’s just a low census…So I think just the sheer fact that there’s…not very many black female anesthesiologists is a problem in and of itself and that [we] should actively recruit and…be intentional about making black female physicians a priority.” (FG9)	Accommodate barriers
“I feel it has to be something optional.” (FG9) “I was gonna say, give time, like time to the mentee to especially like junior faculty with like higher clinical commitments. Just providing time for this is, I think, very important. You know, oftentimes I can’t even remember the amount of times that you’re like back and forth in an email chain about trying to coordinate a time when I can get coffee…and it’s like impossible. I think it’s important that it’s optional.” (FG9) “[Faculty] want to be successful, but I’m not sure they actually want to put in the time to be successful at the thing they think they want to do. It kind of goes to like, if they just want to go home after their shift, … mentorship isn’t going to help that. But if it’s somebody who’s, you know, [internally motivated to achieve a particular goal], you might be able to better figure out how to mentor them.” (FG5) “I also think that…the needs of each individual faculty [are] different, but [also] that the needs of faculty at different times in their lives are also different, which is kind of, yeah, it’s just a reality. … [At times in my career, the truth of the matter has been that] I cannot [do anything additional] at this moment, because of everything else that happens in my life, I just cannot. So we have to also normalize that that’s okay. Yeah. So perhaps we just have to normalize that some people want to do things and they want to have mentors, maybe help people figure out, you know, what it is that they want through coaching and offer a sort of a layered approach.” (FG5)	Allow voluntary participation
“I think a pod system might [be] more useful than one-to-one [mentorship], or both might be applicable and useful in different ways. [For example], if you have a senior attending…and then like middle career, and then some people who are just starting and cohort them together, maybe along interests…or maybe across…different interests, that might be very helpful…When you have, say, a group of young attendings who are just starting out, early attendings, they can crosstalk each other a little bit differently than they can going up and down.” (FG2) “I think one of the things about mentorship is the individualism component. But let’s say you redefine it and you don’t say it’s about the individual but it’s a cohort of individuals.” (FG1) “I think we just have to be thoughtful about and maybe more deliberate about how we assign mentors and mentees and make it less about the personalities than about the work that needs to be accomplished between a mentor and mentee.” (FG6) “I have to assume that someone would only want me as a mentor from a career perspective if they envisioned a similar career to mine. Like I won’t be able to advise you effectively on a career targeting clinical excellence in a specific clinical field [that we don’t share]. I can make some guesses, but like I’m probably not the right person to talk to you about that.” (FG8)	Facilitate intentional mentor–mentee matching
“…maybe there’s a way that [meeting timing and structure are a] little bit more formalized and regular…not to make it like another task to complete, but something a little bit more structured in terms of what the expectations are, maybe at least initially for the first year.” (FG4) “in the very beginning, there’s some sort of person [who] sits a faculty down [to discuss their interests and provide an introduction to the] structure of the department. And then [this person, who is very versed in the process can] suggest mentors to them [and suggest] ‘here are some things that you could get involved in within the medical center that will really help sort of your advancement, [and] this is the expectation’…[For me], I felt like those sort of like, expectations weren’t sort of laid out.” (FG4) “There really needs to be like a short set of questions that a beginning mentor-mentee relationship should try to answer initially…and especially like the first merit cycle, there should be a set of discussion points for that mentor and mentee to go over.” (FG6) “It’s helpful if you can mutually agree on some set of expectations for what you want out of it. So you’re holding each other accountable. It shouldn’t be just the mentor that holds the trainee accountable. It should be each person holding the other one accountable, right? I want to finish this project in six months, submit an abstract to the ASA within nine months, and have a paper written up within 12 months because I want that because it’s helpful for me to establish this line of research or I’m applying for a fellowship program. I want it done before I start applying.” (FG6)	Provide meeting structure
“There’s something meaningful about having a protected and more guided conversation that allows people to articulate something perhaps more deep [than the day-to-day frustrations], something that has been weighing on them for a little bit longer, or even naming something that they haven’t been able to verbalize…[So], what can we do to continue on conversations where people feel like they can express themselves, feel like there’s a way to have this minor catharsis, even though it doesn’t fix the larger problem? I think that is as meaningful for keeping people on as having mentorship, et cetera.” (FG1) “[People get into] their own echo chambers, right?...I definitely gravitate towards the same individuals…but I think making that group more diverse, allowing us to have challenging conversations, really can enrich a perspective towards something that could have been bothering one person and not bothering another. …The open invitation, I think is the first place to begin with.” (FG1) “…the coaching program [was] incredibly helpful because our lives mirrored. [My coach was a person with a similar home life as me]. …I think having someone that can understand my experience as a working mom and a dual working family… would help a lot too.” (FG7) “…if you have people that don’t know how to mentor, it doesn’t inspire you to stick around in a place where you’re hoping to pursue a certain career path. You’re going to go somewhere else. …Whereas if you’re in an environment where you really feel inspired, valued, motivated, and appreciated, whoa, now that’s a big deal, right?” (FG6) “I would like to hear more from people that have some educational credentials…that provide me with some reasonable approaches for me to use and apply and experiment with as a mentor. …So getting independent speakers to come and talk to me about, well, what sort of styles and approaches would work if you want to be a mentor? [This person ideally] comes from an educational background or from a business background or from industry, who really is an expert on this, that has the credentialing that can provide you with the ability to figure out how to support mentor and advise someone in terms of their career development. That would be invaluable.” (FG6)	Align with professional development initiatives

**Fig. 1.**
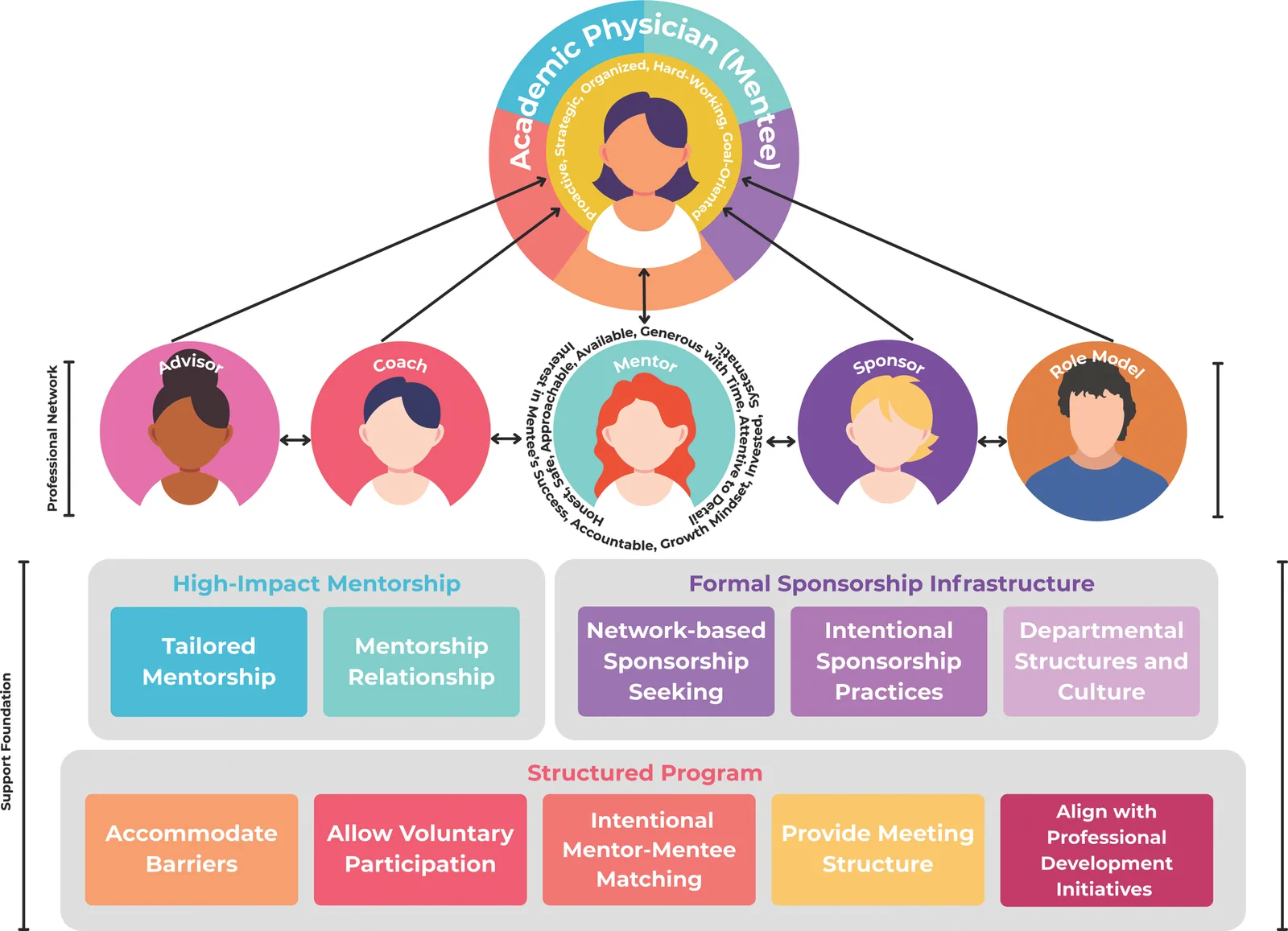
Mentee support pyramid. Themes and subthemes emerging from focus groups are represented as multiple layers of support for the academic physician (mentee), who is the central figure in this process, and the professional support network. The professional network is represented by the row of individuals immediately below the mentee, with the mentor(s) serving the most central role but additional impactful players pictured. The *bidirectional arrows* between individuals in the support network denote that one person can serve more than one of the listed roles or move in and out of the listed roles depending on time and need, whereas the *unidirectional arrows* denote that one individual can also serve one distinct role. The *bidirectional arrow* between mentee and mentor represents the bidirectional mentorship relationship. The descriptors surrounding both the mentee and mentor are characteristics noted as important by study participants.

## Why Is Mentorship Important in Academic Anesthesiology?

Academic anesthesiology faculty are working in a changing landscape with numerous professional and personal stressors that contribute to difficulty achieving traditional academic success and likely influence the high intent to leave academic practice.^18,23,24^ One research faculty explained their perspective on the importance of mentorship and departmental support in their decision to stay in academic medicine:

“I know a lot of [relatively junior] people who have left [the department] who had a lot of potential…and should have probably stayed in academics, but for whatever reason, like they had childcare demands or they just weren’t getting the data [and] mentorship [they needed], opportunities were missed to help those people like bridge that gap. And I feel like I kind of had moments in my career where, you know, I had my kids and then…I had a sick family member and [there were] a lot of moments where I probably could have left research. And it took a lot of mentorship from my primary mentor, but also just like the department recognizing those stressors on my life and giving me a little bit of a break in terms of the productivity while I was taking care of those things so that I could come back.” (FG8)

To maximize the impact of mentorship, participants discussed the need to *tailor mentorship* to both career stage and the professional or personal domain for which mentorship is needed.

Participants noted that the type and character of the guidance needed from a mentor change with career stage and professional goals. For example, new faculty benefit from having designated individual(s) available for practical guidance as they adjust to a new system. Early career faculty require concrete direction on tailoring their professional activities to their individual goals and promotion criteria. Mid-career faculty can use mentorship to develop a broader view of their career trajectory and professional identity. An established-career woman described how mentorship needs change in the later stages of one’s career:

“I don’t think you ever make the transition away from being a mentee, [but] I’m more deliberate about [the way I seek advice] and who I go to…[I] always think I should [continue to seek mentorship] if I want to continue to grow, if that makes sense.” (FG5)

In addition, participants emphasized the need for multiple mentors with distinct areas of expertise to target different professional and personal domains. Some areas that may necessitate mentorship include: clinical work, career or promotion, research content or methods, and navigating personal stressors. An early career woman said:

“We don’t have to just stick with one mentor,…we can have [many mentors] for different reasons – like one for research, one for clinical [work], one who is your person to reach out to [for advice], one person who knows [the institution and] how to navigate the system, [and a sponsor who can pass on] a leadership role when they’re done with their [term].” (FG1)

Different mentorship needs were also highlighted for different individuals. Researchers noted the difference between a “career” mentor, who can provide guidance on professional activities and promotion, and a “content” mentor, who has specific expertise in one’s research methods or content area. For parents or caregivers, participants expressed a desire for guidance on navigating caregiving stress and their careers simultaneously. Individuals from underrepresented groups highlighted the impact of being able to seek mentorship from someone with a shared background or experience or from someone willing to understand the impact of the individual’s unique experience on their career.

## How Should a Mentee Identify and Select an Appropriate Mentor?

The process of establishing a successful mentorship relationship was frequently described as organic, spontaneous, and self-determined. Participants expressed the need for a genuine connection to form between mentor and mentee, which often starts from a place of curiosity about shared experiences or interests. Often, the mentor is a figure the mentee wishes to emulate in some aspect of their career or life and matches well with their personality; thus, such pairings happen best on a mutually voluntary basis. These relationships were frequently described as *organic*. In addition, some participants stressed the need for a mentor to be “safe,” with no direct consequences for their career, to reduce pressure perceived from the relationship. An established-career man said:

“I feel like mentorship [grows] organically out of some sort of group identity. Because then…we have a common language, we can start to talk about, like, you know, ‘What do you do?’ ‘What are you interested in?’ ‘Where do you want to go?’ If it’s somebody…who I just really have no connection with, it’s a lot harder to establish that relationship – you can do it, but it’s harder.” (FG6)

## What Essential Characteristics Should a Good Mentor Possess?

Based on participant experiences, characteristics of an effective mentor include honesty, safety, approachability, availability and/or generosity with time, attention to detail, use of a systematic approach, genuine interest in the mentee’s goals and success, accountability, growth mindset, and investment in mentorship (fig. 1). The mentor’s ability to establish a connection, demonstrate their commitment, and show investment in the mentee’s career was seen to be of utmost importance. Further, formal training or education in effective mentorship was deemed necessary so mentors can provide direct, actionable, effective, and context-specific guidance.

## What Should a Mentor Expect from a Mentee?

Mentees and mentors alike underscored that the mentee role is active rather than passive, although younger mentees may require education to this effect. Mentees benefit from being proactive, strategic, organized, hard-working, self-motivated, and goal-oriented (fig. 1). They should view the mentor–mentee relationship as a bidirectional partnership, primarily driven by the mentee but with benefits for both the mentor and the mentee. Further, mentees should look for opportunities to join groups where community and mentorship or sponsorship opportunities exist.

Mentors emphasized the importance of the mentee taking initiative to initiate regular contact to ensure the mentor’s guidance is truly desired. Mentors viewed a productive relationship as a collaborative partnership, rather than operating in a relational hierarchy. They expressed appreciation for self-motivation and persistence from the mentee to drive the ongoing relationship and also acknowledged the time commitment required to be an effective mentor. One established researcher explained their approach to being a mentor:

“I recognize the things that got me to where I am [required standing on the shoulders of those who came before me]. So I’m mindful of that and I understand the need to help more junior people. I don’t tend to go run to people and say, well, do you need help? I expect people to come to me and ask me if I would provide help. And invariably I do…If you’re imposing on somebody and saying, look, I can help you, then they feel obliged to use you. That’s probably not the way to go.” (FG8)

## How Can an Effective Mentorship Relationship Be Developed, Nurtured, and Sustained?

Establishing and cultivating a meaningful relationship was viewed as essential to high-impact mentorship. Investment from both the mentor and mentee, described as “leaning in,” was seen as a critical part of building and sustaining a mutually productive relationship, as was a growth mindset among both parties through continuing a bidirectional conversation and seeking feedback from each other. Relationships with a genuine connection may ultimately evolve or even dissolve as mentorship needs change over time.

## How Does Sponsorship Complement Mentorship?

Sponsorship was viewed as a key counterpart to mentorship in ensuring career advancement and fulfillment, particularly for those in early- and mid-career. Less established faculty discussed the need to obtain sponsorship to achieve academic success. For example, an underrepresented individual said:

“[My mentor] has that individual push to want to seek people out and offer not just mentorship, but sponsorship, which at my stage is what’s so important because I’m not connected. So I can’t be putting out these papers and getting on these grants and things like that unless people are helping me out.” (FG9)

## What Intentional Practices Are Recommended for Seeking and Offering Sponsorship?

Participants discussed individual strategies for both attaining and providing sponsorship as well as organizational strategies for promoting sponsorship, discussed further below.

### Network-based Sponsorship Seeking

Participants described obtaining sponsorship as an active process that requires leveraging personal and professional networks, engaging in hard work to demonstrate value, and looking beyond immediate departmental boundaries for sponsorship. Casting a wide net to identify sponsors both within and outside the organization was recommended.

### Intentional Sponsorship Practices

Participants described effective sponsorship as a deliberate process involving actively creating opportunities for others while “clearing the lane” for other faculty to take on new professional opportunities. They emphasized the need to ensure that sponsorship opportunities are distributed equitably across individuals. An established-career man said the following about his approach to intentional sponsorship:

“[Early in my career], there were probably opportunities that passed by me that I could have been, you know, pushed towards. And so I try very hard [to do this] for junior faculty and residents and fellows – [to give them] unsolicited advice or…encourage them, and then…on the back end, drop a name in someone’s ear about a potential person that might be a good fit for a position or opportunity within the department.” (FG6)

### Departmental Structures and Culture as Sponsorship Enablers

Participants highlighted the critical role of departments, institutions, and organizations in promoting sponsorship by developing supportive infrastructure, encouraging innovative sponsorship practices, and promoting culture change. These efforts were seen as essential for embedding sponsorship into departmental norms and creating an environment where sponsorship can thrive.

## How Should an Institution or Department Establish, Govern, Evaluate, and Continuously Improve a Formal Mentorship Program?

Participants highlighted the need to formally prioritize mentorship and sponsorship by offering a structured program. The following quote from a mid-career woman explains the rationale:

“…At least the first couple of years, when I was full or near full clinical commitment, you really just have to put your head down and get the work done and there’s nothing left…When there wasn’t a system that could…lower the activation energy at the end of the day when I had no energy and quite frankly, almost left quite a few times because I felt like, ‘what am I doing here?’ [I felt even more lost. Once I] started to like carve out some time where I could work on some of my own projects…that’s kind of what kept me here.” (FG3)

Participants offered the following guidance on potential program organization: (1) accommodate barriers, (2) allow voluntary participation, (3) facilitate intentional mentor–mentee matching, (4) provide meeting structure, and (5) align with professional development initiatives. Further description of these suggestions is provided in figure 2. Figure 3 provides an overview of a candidate mentorship program structure as an example of a formal program that could be studied further. The program pictured incorporates both ideas that came directly from focus group participants and synthesis of participants’ suggestions into potential design elements, denoted with asterisks.

**Fig. 2.**
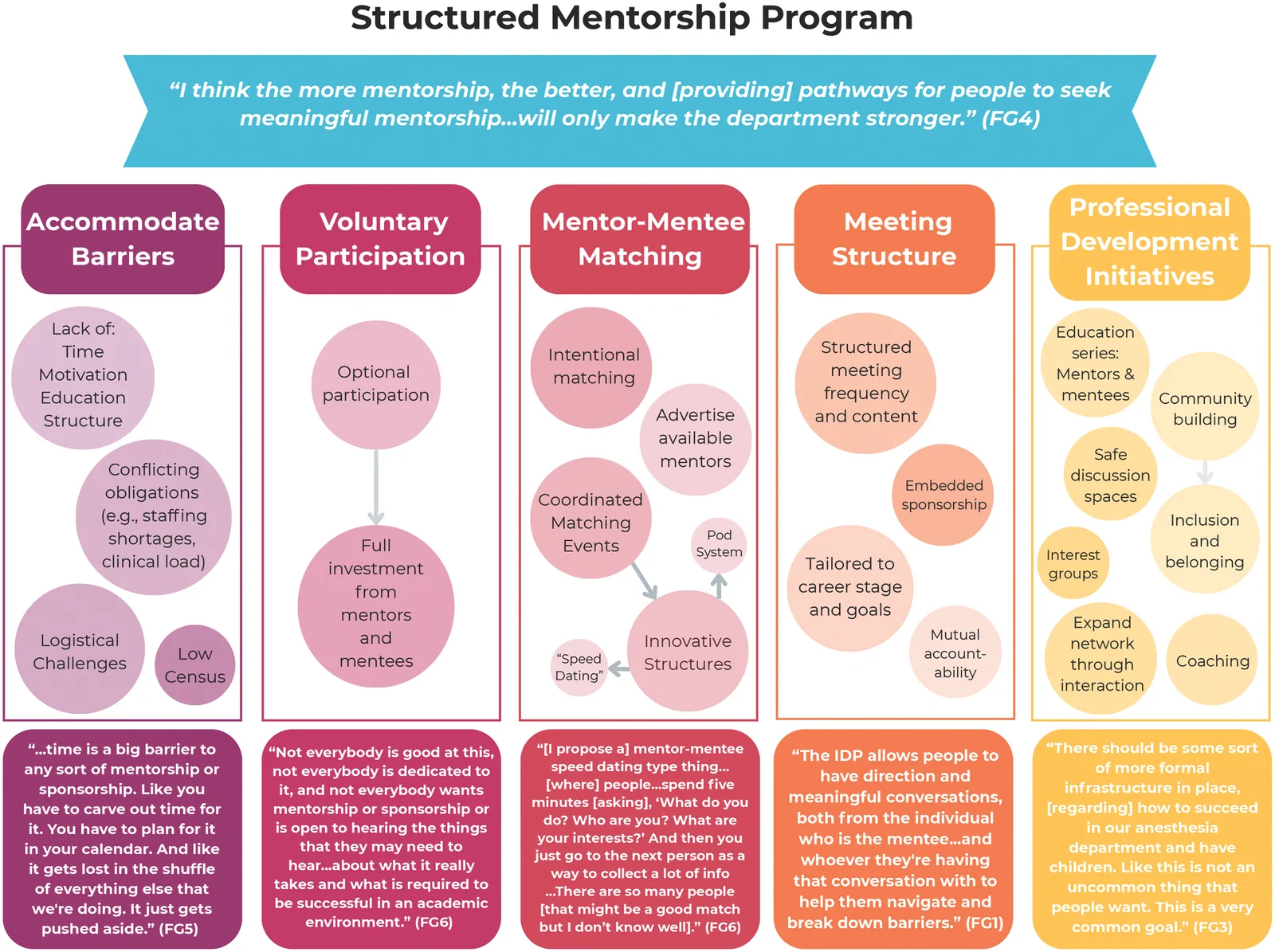
Subthemes of theme 3: Offer a structured program. The five subthemes of theme 3 are described: accommodate barriers, allow voluntary participation, facilitate intentional mentor–mentee matching, provide meeting structure, and align with professional development initiatives. An exemplar quote for each subtheme is provided. The *circles* under each subtheme provide further detail on the rationale and/or suggestions provided by participants. Barriers to mentorship and sponsorship emerging in the data include: lack of time, motivation, education, and structure; conflicting obligations related to high clinical volume or home life; logistical challenges of scheduling meetings between busy individuals; and a “low census” of mentors for underrepresented groups. These barriers should be acknowledged, and creative ways to work around them should be developed. Voluntary participation was suggested to ensure that both mentor and mentee participants are invested in program participation and outcomes. Intentional, individualized matching between mentors and mentees was viewed as a critical component of a mentorship program. A resource to make potential mentees aware of individuals with mentorship interest and expertise was desired. Coordinated events, potentially with innovative structures such as mentorship “speed dating,” could increase access to potential mentors. Other innovative mentorship structures to consider include a pod system, which would allow for both peer mentorship and community. Participants desired structured meeting content to guide meaningful mentoring discussions occurring at a set frequency. They also desired mentorship tailored to their career stage and goals, with sponsorship opportunities embedded into the mentorship program. Aligning these mentorship efforts with a parallel set of initiatives aimed at professional development, including education for both mentors and mentees, safe spaces to discuss potentially sensitive topics with integrated community building to increase inclusion and belonging, opportunities to expand one’s network, and formalized coaching opportunities, was deemed helpful. FG, focus group; IDP, individual development plan.

**Fig. 3.**
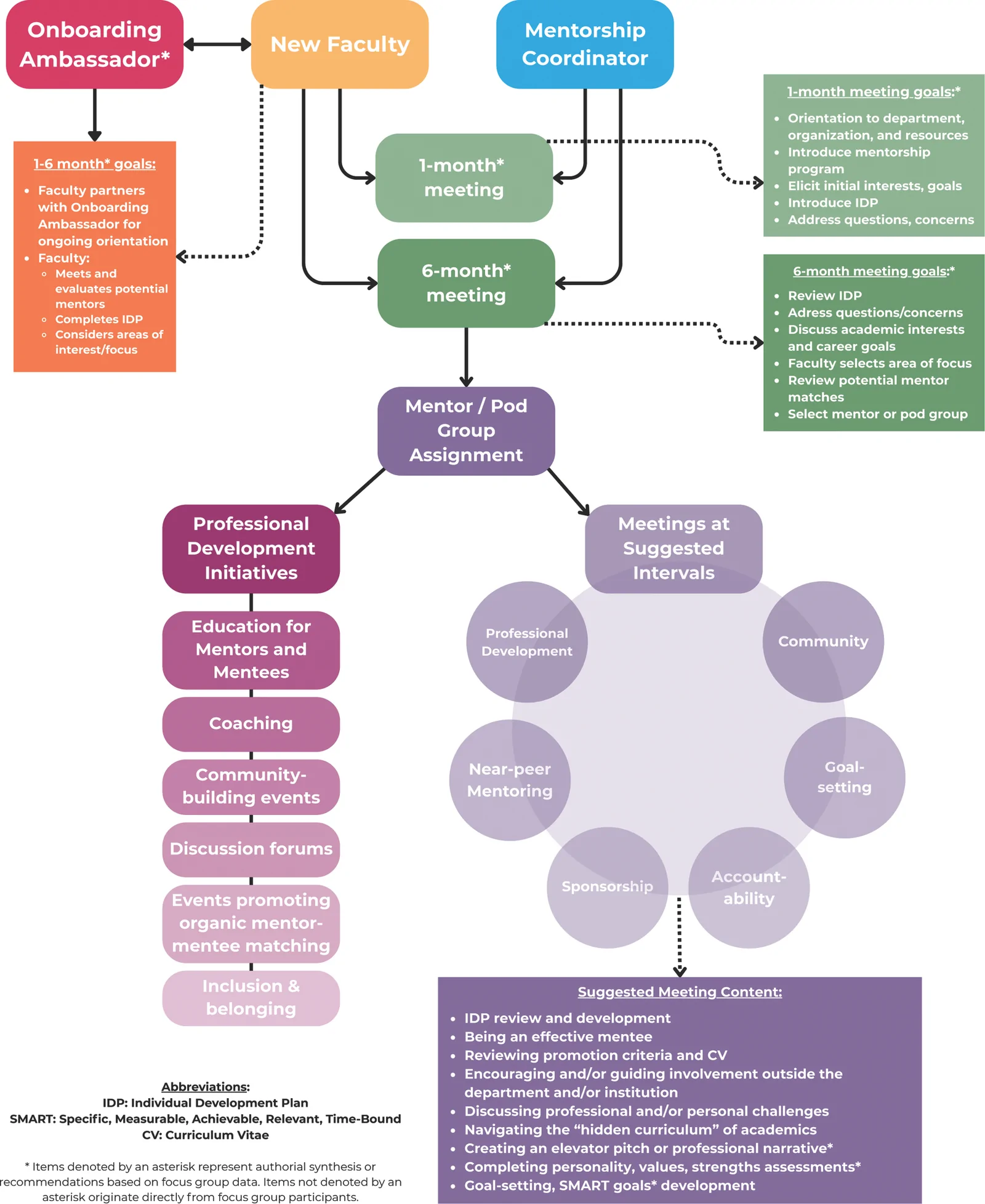
Candidate mentorship program structure. A potential formal mentorship program structure stemming from both focus group participants’ direct suggestions and synthesis of principles raised in the focus group process by authors (denoted by *asterisks*) is presented. In this theoretical program, new faculty are connected with a departmental professionalism leader or “Mentorship Coordinator” to introduce the faculty to the department’s mentorship structure and begin discussion of the faculty’s professional interests. Meetings between the new faculty and Mentorship Coordinator would occur at suggested intervals of 1 month and 6 months, each conducted with prespecified goals. Concurrently, the faculty would be paired with a peer “Onboarding Ambassador” to provide guidance for the day-to-day professional or logistical questions that arise in the faculty’s new role. At an interval of approximately 6 months after onboarding, which would allow enough time for orientation to the department and introduction to potential mentors to occur, the new faculty member would be assigned to a mentor and/or pod group based on their preferences and professional interests. They would then enter the department’s structured mentorship program on a voluntary basis, which would include regular mentorship meetings with predefined discussion content or meeting goals, as well as a parallel and complementary professional development series.

## Discussion and Literature Review

In this article, academic anesthesiology faculty spanning diverse career stages reflect on the critical role of mentors, mentorship, and sponsorship in career development and professional fulfillment. While many experiences with mentorship and sponsorship were expressed in a positive light, negative experiences reported by faculty were also a formative component of the themes identified. Participants characterized “high-impact” mentorship as targeting guidance to appropriate career milestones (*i.e.*, the right kind of mentorship at the right time) and domains of personal and professional life (*i.e.*, the right mentor for the right problem). Key features intrinsic to a productive mentoring relationship were organic relationship formation, helping mentors be effective in their role, and encouraging mentees to be active in their own professional advancement.

Focus group participants suggested principles important for: (1) elevating high-impact mentorship, (2) formalizing sponsorship infrastructure, and (3) implementing a structured program incorporating modern organizational constructs. While proposed program elements deserve further study before implementation, participants desired tailored mentorship to career-stage and content-area needs, mentor–mentee pairings that foster organic relationship formation, and education for mentors and mentees on effective mentorship strategies and proactive mentee behaviors. Participants suggested elevating sponsorship by using one’s network to actively seek sponsorship opportunities, providing sponsorship intentionally, and focusing on departmental or institutional infrastructure and culture change to enable sponsorship. According to faculty perspectives, structured mentorship programs should focus on eliminating barriers, allowing voluntary participation, facilitating intentional mentor–mentee matching, providing a structured meeting format, and aligning with professional development initiatives. The novelty of this work lies in the synthesis of structures proposed across career stages and identity-specific groups, which captures the priorities of individuals with diverse lived experiences within a single department. The potential design principles for mentorship and sponsorship infrastructure arising from this study generate hypotheses requiring further testing.

The mentor–mentee relationship emerged thematically in various ways, suggesting that mentor–mentee compatibility is critical to constructive relationships. Prior authors have reported that mentees characterize mentoring relationships that form organically within facilitating organizational support systems as higher in satisfaction and perceived success.^1,25,26^ Furthermore, successful mentorship relationships revolve around mutual respect, clear expectations, reciprocity, personal connection, and shared values, whereas failed mentorship relationships are more likely to involve communication challenges, personality differences, a lack of sincere interest in the mentee’s success, and inexperienced mentors.^27^ During the initiation and cultivation phases of the mentorship relationship, psychological safety is also key.^1^ Given evidence in the literature that women and members of underrepresented groups may face additional challenges in the mentoring relationship,^28^ members of vulnerable populations may prefer mentors who share similar backgrounds or characteristics; however, both in our data (n = 20 women, parents, and underrepresented in medicine faculty) and in the literature, the investment of the mentor is prioritized over their actual gender or cultural background.^29^

The need for knowledge and training in mentorship best practices was frequently discussed in focus groups. The importance of mentorship expertise has also been acknowledged by the National Institutes of Health, which now requires that mentors of T32 award recipients receive formal mentor training and periodic knowledge updates.^30^ Numerous theories and conceptual models exist that can help mentors guide their mentees toward achieving their goals. One example is the Daloz model of balancing support and challenge, which argues for mentorship practices that couple high support with high challenge to empower the mentee, foster a growth mindset, and promote progress.^1^ Similarly, mentorship rooted in the tenets of Ecological Psychology, a framework that considers the interplay between an individual’s career aspirations and environmental influences on their ability to actually achieve their goals, can allow both mentee and mentor to focus an individual’s efforts on the most achievable goals in the context of their current personal and professional life.^2^ Thus, mentors with a theoretical understanding of mentorship best practices may be better equipped to guide their mentees toward their goals. Further, characteristics such as honesty, sincerity, active listening, and empathy have been described as desirable in a mentor,^29^ so providing formal training to mentors in the social-emotional aspects of mentorship may also be beneficial. In our data, instances of mentorship for primary gain emerged; several suggested design components of a mentorship program, including mentor training, may have prevented or alleviated such experiences.

Mentorship relationships have been described as bidirectional,^1^ reinforcing the active role of the mentee. Mentees should intentionally seek out mentors with desired personal or professional characteristics, develop specific goals with a clear timeframe and checkpoints, understand their own strengths and limitations, and be willing to step outside of their comfort zone^1^—elsewhere called “taking the driver’s seat.”^31^ Some established-career participants in this study acknowledged that they had not been ideal mentees in their earlier career stages because they lacked understanding of the skills required to engage successfully in the mentoring process. Where authors encountered such dissenting remarks or other negative experiences with mentorship, programmatic design principles to address the participant’s experience were suggested. For example, inspired by the “terrible mentee” in FG5 and other participants’ observations that mentors may feel exploited by a one-sided relationship, providing formal education to mentees so they may effectively execute their roles and responsibilities in their own development was a suggested element of a mentorship program. Similarly, the concerns from participants that described “predatory” mentorship were addressed using the suggestions of voluntary participation, intentional mentor–mentee matching, mentor training, and fostering positive attributes in mentors.

Sponsorship is a vital complement to mentorship—in the words of Ayyala *et al.*, “Mentorship Is Not Enough.”^32^ The need for formal sponsorship infrastructure highlighted in this study was characterized by strategic sponsorship-seeking, intentional practices for providing sponsorship, and structures embedded within a departmental culture aimed at enabling sponsorship. These priorities are reflected in the literature as well. Compared with mentorship, sponsorship often occurs more intermittently but with a greater focus on a particular professional development or advancement opportunity, with the overall goal of enhancing an individual’s visibility and professional networks.^8,32^ These relationships differ significantly: sponsorship requires the recipient to earn the sponsor’s respect and trust, as the sponsor leverages their own social capital to create a professional opportunity. Sponsorship recipients, therefore, must demonstrate behaviors or traits that would be considered worthy of sponsorship, such as aptitude for the task, promise of success, follow-through, and dependability, among others.^32^ Prior authors have proposed that deliberately using sponsorship for career advancement is an important strategy for early and mid-career faculty.^8,32^ Furthermore, sponsorship can be integrated into departmental culture or formal career development structures.^8^ It is widely reported that women tend to be “under-sponsored” and may be excluded from informal networking opportunities, which contributes to ongoing gender disparities; thus, intentionally creating sponsorship and networking opportunities for women may be a key to balancing the ongoing gender inequity reported in academic medicine.^7,8,33,34^

Focus group participants consistently desired structure to increase the impact and utilization of both mentorship and sponsorship. Many such successful structures have been previously described, including a facilitated peer mentoring program,^35^ a career-building framework intended for use in national working groups,^36^ a research scholars’ program,^37,38^ and an academic promotion support project including embedded coaching.^39^ One such example in anesthesiology is the Women’s Empowerment and Leadership (WELI) program for pediatric anesthesiologists, which has reported positive outcomes including making personal connections, implementing new projects, achieving career advancement, improving work-life integration, and building confidence.^40–42^ However, other authors report that the introduction of a junior faculty professional development program led to increased burnout and decreased quality of life by the end of the program, likely due to increased workload and challenges with work-life integration^43,44^; thus, the impact of such programs on participant well-being must be carefully considered in their design. In this study, participants suggested that a mentorship program should be comprehensive, facilitating matching between compatible mentors and mentees, integrating novel structures beyond the traditional dyadic mentorship relationship, providing structured content to guide mentorship discussions, and aligning mentorship with complementary professional development efforts to encourage open discourse and community building. Many of these suggestions are well-aligned with previously published studies. Newer models of mentorship involving team, peer, and network structures have gained traction in the literature.^1^ Novel constructs such as “speed mentoring” and peer- and group mentoring have emerged in response to the notion that no single mentor is likely to meet all of a mentee’s needs.^1^ Peer group mentoring programs have demonstrated a positive impact on self-efficacy in career advancement, research, and mentoring, and higher cognitive empathy, among other career-related skills.^44,45^ Ideally, such a program would allow an individual to develop a mentorship network comprised of many mentors with diverse areas of expertise and departmental or institutional affiliations.

While mentors may act as coaches within the purview of their mentorship responsibilities, dedicated coaching has also been incorporated into successful mentorship programs.^39^ Coaching is often a shorter-term, performance-driven relationship that can be used within any career pathway.^46^ The coaching role is gaining popularity among clinician-educators, as it encourages learners to set goals, engage in self-reflection, and make changes that maximize their performance. Many positive impacts of coaching have been previously described in medical education^47–49^ and at the faculty level^50–52^; therefore, great potential exists to formally integrate coaching practices into a structured program for faculty.^53^

### Acknowledgement of Limitations

The authors acknowledge that mentorship and sponsorship principles derived from this study come from a small number of participants at a single institution, which may limit the transferability of the study’s findings. However, the diverse clinical practice of study participants across six separate clinical sites with distinct patient populations, work environments, and leadership structures, along with the inclusion of faculty across career stages and those belonging to underrepresented groups, increases the likelihood that their perspectives reflect broader lived experiences shared by faculty outside the study institution. Focus group studies risk inherent biases stemming from peer influence; attempts were made to limit bias related to the focus group study design by assigning focus groups by career stage and gender, which increased participants’ comfort levels in engaging in candid discussion. Selection bias may have been introduced by selecting participants from those who had completed a previously described department-wide survey, potentially shifting the sample toward a more engaged population. As a result, more disengaged or peripheral faculty’s voices may not have been fully represented.

It is also important to note that an all-women coding team completed the analysis, including that of the all-men focus groups. The team’s analytic perspectives were shaped by lived gendered experiences and social positions, which may have influenced the salience of the identified themes and attributed meanings. Throughout the analytic process, the team engaged in discussions to examine how individual positionality may have affected interpretation and remained attentive to the risks of misinterpreting men’s accounts through the lens of women’s experiences. However, the outsider perspective was valuable for allowing identification of implicit norms and gendered expectations that may have been less visible to those whose positionality was more concordant with the participants’. Instead of viewing gender concordance as a prerequisite for valid interpretation, authors viewed the analytic process as one in which meaning was necessarily co-constructed between the participants and the researchers’ interpretations. As such, findings are presented with the acknowledgement that alternative results may have emerged from a coding team consisting of different gender and professional identities.

## Conclusions

Academic anesthesiology faculty spanning all career stages identified their need for high-impact mentorship from trained mentors tailored to individual career development; accessible sponsorship opportunities; and a formal mentorship program incorporating novel structures, discussion guides, education for mentors and mentees, and attention to growing inclusion and belonging. Mentors, sponsors, and coaches all play important roles in facilitating development of fulfilling and sustainable careers in academic anesthesiology, although mentees should be proactive and systematic to intentionally drive their own careers toward their goals.

## Acknowledgments

The authors would like to extend their deepest gratitude to the Department of Anesthesia and Perioperative Care at the University of California, San Francisco, San Francisco, California for providing departmental research funding to support this work.

## Research Support

Dr. Sinskey and Dr. Schwartz receive National Institutes of Health (Bethesda, Maryland) grant funding from the National Institute of General Medical Sciences (FAIN#:R25GM153811). Support was also provided by departmental sources.

## Competing Interests

Dr. Schwartz receives royalties from McGraw Hill (New York, New York) and consults for the Society of Pediatric Anesthesia (Richmond, Virginia). The other authors declare no competing interests.

## Supplemental Digital Content

Focus group interview guides, Supplemental Digital Content 1: https://links.lww.com/ALNO/A28.

## Supplementary Material
