# Supplementary material for: Mentees’ Perspectives on Mentorship and Sponsorship in Academic Anesthesiology: Findings from a Single-institution Focus Group
Source: Anesthesiol Open. 2026 Sep 30;1(3):e0033. doi: 10.1097/ao9.0000000000000033 (PMC13650749; doi:10.1097/ao9.0000000000000033)
Supplement: Supplementary file 1 [file ao9-1-e0033-s001.pdf]

Introduction (all focus groups)

Moderator intro: Hello, and thank you for participating in this focus group. As you know, our goal is to hear faculty members' perspectives on career success in our department, specifically focusing on mentorship and sponsorship. I have about 15 questions to get through, and I want to respect the 1-hour time window we've set aside. So, there may be points in conversation where, in the interest of time, I need to move forward with the next question. This doesn't mean I'm not interested in what you're saying – I would be happy to speak more after the focus group concludes.

What questions do you have for me before we begin?

Early-Career Women

I'm going to start with some questions to gather basic information:

1. How long have you been at UCSF?
2. Which site(s) do you practice at?
3. Are your professional responsibilities mostly clinical, mostly academic/administrative, or somewhere in between?
4. Briefly describe the kind(s) of non-clinical work you do.

Next, I'd like to hear about your experiences as a faculty member.

5. What do you value about being a faculty member in the Department of Anesthesia & Perioperative Care at UCSF?
6. What are the biggest challenges facing new faculty members?
    a. What has helped you succeed?
    b. What would have helped you as a new faculty member?
7. What makes academic work meaningful or worth pursuing to you?
    a. How have you approached finding academic or scholarly work that is worth pursuing?
8. When considering a new academic opportunity or leadership position:
    a. What are factors that would encourage you to apply for the position?
    b. What are factors that would be detractors?
9. Next, I'm going to ask some questions about allyship, mentorship, and sponsorship. To make sure the terminology is clear, here are the definitions we'll be using.
   <u>Ally</u>*: A trusted person who supports you and helps you achieve your goals*
   <u>Mentor</u>*: Someone who provides advice and/or guidance on career and work*
   <u>Sponsor</u>*: Someone who creates or connects you with career-advancing opportunities by using their own influence or social capital.*
    a. Tell me a bit more about your experience with allyship, mentorship, and sponsorship in our department.
    b. Do you currently have an ally at work? A mentor? A sponsor?
    c. How have you approached finding mentors/sponsors in the department?
        i. Have you ever actively sought mentorship/sponsorship?

   d. Describe the qualities or practices of someone who has been an effective mentor or sponsor to you in the past.
   e. What have been the barriers to finding a mentor or sponsor and developing effective mentorship relationships?
10. If our department were to design a structured mentoring program for faculty members, what types of components would be most helpful to you?
    a. What are the specific components that would help early career faculty members?
11. Do you think things are different for women and men in our department? How?
    a. How does being a woman impact the clinical and academic work you do in our department?
    b. What specific components of a mentorship program would help women succeed?
12. How could our department do a better job in helping people to succeed within specific career pathways (for example: education, QI, research)?
13. What could the department do to elevate the accomplishments of women in our department?
14. What do you think are the major threats to retaining early-career faculty in our department?
15. What question do you wish I had asked today? [If they say "nothing, that was good" try again by prompting: "what question do you wish I had asked that I didn't?"

Early-Career Men

I'm going to start with some questions to gather basic information:

1. How long have you been at UCSF?
   2. Which site(s) do you practice at?
   3. Are your professional responsibilities mostly clinical, mostly academic/administrative, or somewhere in between?
   4. Briefly describe the kind(s) of non-clinical work you do.

Next, I'd like to hear about your experiences as a faculty member.

5. What do you value about being a faculty member in the Department of Anesthesia & Perioperative Care at UCSF?
6. What are the biggest challenges facing new faculty members?
   a. What has helped you succeed?
   b. What would have helped you as a new faculty member?
7. When considering a new academic opportunity or leadership position:
   a. What are factors that would encourage you to apply for the position?
   b. What are factors that would be detractors?
8. What makes academic work meaningful or worth pursuing to you?
   a. How have you approached finding academic or scholarly work that is worth pursuing?
9. Next, I'm going to ask some questions about allyship, mentorship, and sponsorship. To make sure the terminology is clear, here are the definitions we'll be using.

<u>Ally</u>: A trusted person who supports you and helps you achieve your goals
<u>Mentor</u>: Someone who provides advice and/or guidance on career and work
<u>Sponsor</u>: Someone who creates or connects you with career-advancing opportunities by using their own influence or social capital.

   a. Tell me a bit more about your experience with allyship, mentorship, and sponsorship in our department.
   b. Do you currently have an ally at work? A mentor? A sponsor?
   c. How have you approached finding mentors/sponsors in the department?
      i. Have you ever actively sought mentorship/sponsorship?
   d. Describe the qualities or practices of someone who has been an effective mentor or sponsor to you in the past.
   e. What have been the barriers to finding a mentor or sponsor and developing effective mentorship relationships?

10. If our department were to design a structured mentoring program for faculty members, what types of components would be most helpful to you?
    a. What are the specific components that would help early career faculty members?
    b. How would it land with you If there were specific mentorship tracks targeted toward women or underrepresented groups to which you do not belong?
11. How could our department do a better job in helping people to succeed within specific career pathways (for example: education, QI, research)?
12. Do you think things are different for women and men in our department? How?
    a. How does being a man impact the clinical and academic work you do in our department?
13. What could the department do to showcase the contribution clinicians bring to the department? In your opinion, would it be worthwhile pursuing this avenue?
14. What do you think are the major threats to retaining early-career faculty in our department?
15. What question do you wish I had asked today? [If they say "nothing, that was good" try again by prompting: "what question do you wish I had asked that I didn't?"

Mid-Career Women

I'm going to start with some questions to gather basic information:

1. How long have you been at UCSF?
2. Which site(s) do you practice at?
3. Are your professional responsibilities mostly clinical, mostly academic/administrative, or somewhere in between?
4. Briefly describe the kind(s) of non-clinical work you do.

Next, I'd like to hear about your experiences as a faculty member.

5. Why do you choose to stay a faculty member in the Department of Anesthesia & Perioperative Care at UCSF?
6. What are the biggest challenges facing mid-career faculty members?

7. How have you approached finding academic or scholarly work that is meaningful to you?
    a. What makes academic work meaningful?
    b. When you hit moments where your work has felt less meaningful, what strategies have you used?
8. Tell me a bit about the mentorship/sponsorship you've received throughout your career, where mentorship is defined as "*advice and/or guidance on career and work"* and sponsorship is defined as "*creation of or connection with career-advancing opportunities using influence or social capital."*
    a. Do you currently have a mentor? Sponsor?
    b. How have you approached finding mentors/sponsors in the department?
        i. Have you ever actively sought out mentorship/sponsorship?
    c. Describe the qualities or practices of someone who has been an effective mentor to you in the past.
    d. What have been the barriers to finding a mentor or sponsor and developing effective mentorship relationships?
9. If our department were to design a structured mentoring program for faculty members, what are the components that would be most helpful to you?
    a. What are the specific components that would help mid-career faculty members?
    b. What are the specific components that would help women?
10. How could our department do a better job in helping people to succeed within specific career pathways (for example: education, QI, research)?
11. What could the department do to elevate the accomplishments of women in our department?
12. What do you wish you had known that you know now when you first joined faculty?
13. What question do you wish I had asked?

Mid-Career Men

I'm going to start with some questions to gather basic information:

1. How long have you been at UCSF?
2. Which site(s) do you practice at?
3. Are your professional responsibilities mostly clinical, mostly academic/administrative, or somewhere in between?
4. Briefly describe the kind(s) of non-clinical work you do.

Next, I'd like to hear about your experiences as a faculty member.

5. Why do you choose to stay a faculty member in the Department of Anesthesia & Perioperative Care at UCSF?
6. What are the biggest challenges facing mid-career faculty members?
7. How have you approached finding academic or scholarly work that is meaningful to you?

a. What makes academic work meaningful?
b. When you hit moments where your work has felt less meaningful, what strategies have you used?

8. Tell me a bit about the mentorship/sponsorship you've received throughout your career, where mentorship is defined as "*advice and/or guidance on career and work"* and sponsorship is defined as "*creation of or connection with career-advancing opportunities using influence or social capital."*
    a. Do you currently have a mentor? Sponsor?
    b. How have you approached finding mentors/sponsors in the department?
        i. Have you ever actively sought out mentorship/sponsorship?
    c. Describe the qualities or practices of someone who has been an effective mentor to you in the past.
    d. What have been the barriers to finding a mentor or sponsor and developing effective mentorship relationships?
9. If our department were to design a structured mentoring program for faculty members, what are the components that would be most helpful to you?
    e. What are the specific components that would help mid-career faculty members?
    f. How would it land with you If there were specific mentorship tracks targeted toward women or underrepresented groups to which you do not belong?
10. How could our department do a better job in helping people to succeed within specific career pathways (for example: education, QI, research)?
11. What could the department do to showcase the contribution clinicians bring to the department? In your opinion, would it be worthwhile pursuing this avenue?
12. What do you wish you had known that you know now when you first joined faculty?
13. What question do you wish I had asked today? [If they say "nothing, that was good" try again by prompting: "what question do you wish I had asked that I didn't?"

Established-Career Women

I'm going to start with some questions to gather basic information:

1. How long have you been at UCSF?
2. Which site(s) do you practice at?
3. Are your professional responsibilities mostly clinical, mostly academic/administrative, or somewhere in between?
4. Briefly describe the kind(s) of non-clinical work you do.

Next, I'd like to hear about your experiences as a faculty member.

5. Why do you choose to stay on faculty in the Department of Anesthesia & Perioperative Care at UCSF?
6. What are the biggest challenges facing faculty members in your career phase?
7. How have you approached finding academic or scholarly work that is meaningful to you?
    a. What makes academic work meaningful?

   b. When you hit moments where your work has felt less meaningful, what strategies have you used?
8. Tell me a bit about the mentorship/sponsorship you've received throughout your career, where mentorship is defined as "*advice and/or guidance on career and work"* and sponsorship is defined as "*creation of or connection with career-advancing opportunities using influence or social capital."*
   c. How have you approached finding and maintaining mentorship relationships throughout your career?
   d. Have you ever actively sought out mentorship/sponsorship?
   e. Describe the qualities or practices of someone who has been an effective mentor to you in the past.
   f. What have been the barriers to finding a mentor or sponsor?
9. Do you mentor or sponsor others in the department?
   a. If so, how did you make the transition from mentee to mentor?
   b. Did you ever have formal training in how to be a mentor?
      i. If Yes: What kind of training? How did it help?
      ii. If No: Would you pursue mentorship training if available to you? What would it look like in order to be meaningful to you?
10. If our department were to design a structured mentoring program for faculty members, what components do you think would be most helpful to faculty?
   c. How should the department approach engaging established faculty members to serve as career mentors for early- or mid-career faculty members?
   d. What would motivate you or other faculty members to participate as mentors?
   e. What barriers do you see to becoming a mentor to others or expanding your mentorship role?
   f. What domains of life and work do you see as pertinent to a mentorship relationship?
11. How could our department do a better job in helping people to succeed within specific career pathways (for example: education, QI, research)?
12. What could the department do to elevate the accomplishments of women in our department?
   g. What are specific components of a mentoring program that would help women faculty members?
13. What do you wish you had known when you first joined faculty that you know now?
14. What do you think are the major threats to retaining late-career faculty in our department?
15. What question do you wish I had asked today? [If they say "nothing, that was good" try again by prompting: "what question do you wish I had asked that I didn't?"

Established-Career Men

I'm going to start with some questions to gather basic information:

1. How long have you been at UCSF?
2. Which site(s) do you practice at?
3. Are your professional responsibilities mostly clinical, mostly academic/administrative, or somewhere in between?
4. Briefly describe the kind(s) of non-clinical work you do.

Next, I'd like to hear about your experiences as a faculty member.

5. Why do you choose to stay on faculty in the Department of Anesthesia & Perioperative Care at UCSF?
6. What are the biggest challenges facing faculty members in your career phase?
7. How have you approached finding academic or scholarly work that is meaningful to you?
    a. What makes academic work meaningful?
    b. When you hit moments where your work has felt less meaningful, what strategies have you used?
8. Tell me a bit about the mentorship/sponsorship you've received throughout your career, where mentorship is defined as "*advice and/or guidance on career and work"* and sponsorship is defined as "*creation of or connection with career-advancing opportunities using influence or social capital."*
    a. Do you currently have a mentor? Sponsor?
    b. How have you approached finding and maintaining mentorship relationships throughout your career?
    c. Have you ever actively sought out mentorship/sponsorship?
    d. Describe the qualities or practices of someone who has been an effective mentor to you in the past.
    e. What have been the barriers to finding a mentor or sponsor?
9. Do you mentor or sponsor others in the department?
    g. If so, how did you make the transition from mentee to mentor?
    h. Did you ever have formal training in how to be a mentor?
        i. If Yes: What kind of training? How did it help?
        ii. If No: Would you pursue mentorship training if available to you? What would it look like in order to be meaningful to you?
10. If our department were to design a structured mentoring program for faculty members, what components do you think would be most helpful to faculty?
    i. How should the department approach engaging established faculty members to serve as career mentors for early- or mid-career faculty members?
    j. What would motivate you or other faculty members to participate as mentors?
    k. What barriers do you see to becoming a mentor to others or expanding your mentorship role?
    l. What domains of life and work do you see as pertinent to a mentorship relationship?
11. How could our department do a better job in helping people to succeed within specific career pathways (for example: education, QI, research)?

12. What could the department do to showcase the contribution clinicians bring to the department? In your opinion, would it be worthwhile pursuing this avenue?
13. What do you wish you had known when you first joined faculty that you know now?
14. What do you think are the major threats to retaining established faculty in our department?
15. What question do you wish I had asked today? [If they say "nothing, that was good" try again by prompting: "what question do you wish I had asked that I didn't?"

Researchers

I'm going to start with some questions to gather basic information:

1. How long have you been at UCSF?
2. Do you spend more than 50% or your professional time on research?
3. Which site(s) do you practice at clinically?
4. What is your title (Assistant, Assoc, Prof)?

Next, I'd like to hear about your experiences as a faculty member.

5. What do you value about your career as a faculty member and researcher in the Department of Anesthesia & Perioperative Care at UCSF?
6. What are the biggest challenges and/or stressors you face related to your research?
    a. What strategies do you use to overcome/address those challenges?
    b. How do you think these challenges compare to those experienced by faculty members in our department who do primarily clinical work?
7. What are the biggest challenges and/or stressors you face related to your clinical work?
8. How have you approached finding and working with research mentors?
    a. Is there any way you could have been better supported in this process?
9. Describe the qualities or practices of someone who has been an effective mentor to you.
    a. How can we encourage these same qualities or practices in others?
    b. What are the characteristics of a successful mentoring relationship?
    c. How many mentors do you have? In what domains?
    d. What is a domain where you would like to have mentorship but have been unable to find it?
10. Describe your experience working within our department's research infrastructure. What if any improvements could be made?
11. How could a formal mentorship program benefit researchers in our department?
    a. What are the specific components that should be targeted toward researchers or those hoping to cross over into research?
12. Do you experience impostor syndrome? With clinical work, with research, or with both?
    a. What strategies have you used to overcome impostor syndrome?

13. For those of you who identify as women, how does being a woman impact your experience as a researcher in our department?
    a. On a national scale?
14. What do you think are the major threats to recruiting and retaining researchers in our department?
15. What question do you wish I had asked today? [If they say "nothing, that was good" try again by prompting: "what question do you wish I had asked that I didn't?"

Parents

I'm going to start with some questions to gather basic information:

1. How long have you been at UCSF?
2. Which site(s) do you practice at?
3. What is your professional title?
4. Are your professional responsibilities mostly clinical, mostly academic/administrative, or somewhere in between?
5. Briefly describe the kind(s) of non-clinical work you do.

Next, I'd like to hear about your experiences as a faculty member and parent.

6. How many children do you have, and what are their ages?
7. Describe your childcare or school arrangements.
8. How does being a parent impact your experience doing clinical work in our department?
9. How does being a parent impact your experience while doing academic work in our department?
10. How do work challenges impact your home life?
11. Tell me a bit about how you work to balance your work and home responsibilities.
    a. What strategies do you use?
    b. What are the places that are ongoing pain points?
12. How does the department's current approach to scheduling and staffing of clinical work impact you as a parent?
    a. What types of changes to clinical scheduling would improve things for you as a parent?
    b. What types of flexible work arrangements would you like to see as offerings in our department?
13. How does being a parent impact your career trajectory?
    a. How could more structured mentorship and/or sponsorship in our program help parents succeed in an academic career?
    b. What domains of life and work do you see as pertinent to a mentorship relationship?
    c. What are some department-sponsored resources that would help parents?
14. When considering a new academic opportunity or leadership position:
    a. What are factors that would encourage you to apply for the position?

   b. What are factors that would be detractors?
15. What are the major challenges faced by **<u>new</u>** parents (i.e., children age 2 and under) in our department?
16. What could the department do to better support **<u>new</u>** parents? If needed for clarification: around the time of return from parental leave and lactation?
17. What do you think are the major threats to retaining parents in our department?
18. What question do you wish I had asked today? [If they say “nothing, that was good” try again by prompting: “what question do you wish I had asked that I didn’t?”

Underrepresented in Medicine

I’m going to start with some questions to gather basic information:

1. How long have you been at UCSF?
2. Which site(s) do you practice at?
3. What is your academic title (assistant, assoc, prof)?
4. Are your professional responsibilities mostly clinical, mostly academic/administrative, or somewhere in between?
5. Briefly describe the kind(s) of non-clinical work you do.
6. If you feel comfortable sharing, what underrepresented group do you identify with?

Next, I’d like to hear about your experiences as a faculty member.

7. Why do you choose to stay a faculty member in the Department of Anesthesia & Perioperative Care at UCSF?
8. What are the biggest challenges you face related to your work?
9. How does your personal identity/-ies impact your experience while doing clinical work in our department? Academic work?
10. What makes academic work meaningful or worth pursuing to you?
    a. How have you approached finding academic or scholarly work that is worth pursuing?
11. When considering a new academic opportunity or leadership position:
    b. What are factors that would encourage you to apply for the position?
    c. What are factors that would be detractors?
12. Next, I’m going to ask some questions about allyship, mentorship, and sponsorship. To make sure the terminology is clear, here are the definitions we’ll be using.
    <u>Ally</u>: A trusted person who supports you and helps you achieve your goals
    <u>Mentor</u>: Someone who provides advice and/or guidance on career and work
    <u>Sponsor</u>: Someone who creates or connects you with career-advancing opportunities by using their own influence or social capital.
    d. Tell me a bit more about your experience with allyship, mentorship, and sponsorship in our department.
    e. Do you currently have an ally at work? A mentor? A sponsor?
    f. How have you approached finding mentors/sponsors in the department?
        i. Have you ever actively sought mentorship/sponsorship?

        ii. What does a successful mentorship relationship look like to you?

   g. Describe the qualities or practices of someone who has been an effective mentor or sponsor to you in the past.
   h. What domains of life and work do you see as pertinent to a mentorship relationship?
   i. What have been the barriers to finding a mentor or sponsor and developing effective mentorship relationships?

13. At UCSF and in our department, there is an emphasis on diversity, equity, inclusion, and allyship.
    j. What are your thoughts about the alignment between the stated values and your daily experiences? Can you provide some concrete examples?
    k. What do you find meaningful about the work done so far in this realm?
    l. What specific intervention(s) would improve your experience?
14. If our department were to design a mentorship program for faculty members, what are specific resources or components that would be helpful to you?
15. What do you think are the major threats to retaining faculty from underrepresented groups in our department?
16. What question do you wish I had asked today? [If they say “nothing, that was good” try again by prompting: “what question do you wish I had asked that I didn’t?”
